# Mixed-methods evaluation of family medicine research training and peer mentorship in Lesotho

**DOI:** 10.4102/phcfm.v12i1.2387

**Published:** 2020-10-19

**Authors:** Chelsea M. McGuire, Kaitlyn Riffenburg, Sebaka Malope, Brian Jack, Christina P.C. Borba

**Affiliations:** 1 Family Medicine Specialty Training Program, Lesotho-Boston Health Alliance, Leribe, Lesotho; 2 Department of Family Medicine, School of Medicine, Boston University, Boston, United States of America; 3 Department of Global Health, School of Public Health, Boston University, Boston, United States of America; 4 Lesotho-Boston Health Alliance, Maseru, Lesotho; 5 Center for Health System Design and Implementation, Institute for Health System Innovation and Policy, Boston University, Boston, United States of America; 6 Department of Psychiatry, School of Medicine, Boston University, Boston, United States of America

**Keywords:** research training, peer mentorship, Lesotho, family medicine, primary health care

## Abstract

**Background:**

Strengthening primary care research capacity is a priority globally. Family medicine training programmes in sub-Saharan Africa represent an important opportunity to build primary care research; however, they are often limited by insufficient research training and mentorship. Peers can be used to extend research mentorship capacity, but have not been evaluated in this context.

**Aim:**

The aim of this study was to evaluate one family medicine training programme’s research capacity building efforts through a blended research curriculum and peer mentorship.

**Setting:**

Lesotho is a landlocked country within South Africa of approximately two million people. The Family Medicine Specialty Training Programme (FMSTP) is the only accredited postgraduate medical education programme in Lesotho.

**Methods:**

This two-year mixed-methods evaluation used: (1) Likert-scale surveys measuring trainee research confidence, (2) written evaluations by trainees, peers, programme faculty and administrators and (3) in-depth, semi-structured interviews. Survey data were analysed using Friedman and sign tests. Interview and written data were analysed thematically via a mixed inductive-deductive approach using Cooke’s framework.

**Results:**

Family Medicine Specialty Training Programme trainees (*n* = 8) experienced moderate increases in research confidence that were statistically significant. Skill-building occurred primarily via experiential learning. Research was grounded in trainees’ clinical practice and locally relevant. A positive research culture was created, promising for sustainability. We identified infrastructure gaps, including funding and protected time. Peer research mentorship supported trainees’ motivation and provided a safe space for questions.

**Conclusion:**

The FMSTP research curriculum and peer mentorship programme were successful in positively impacting a number of Cooke’s research capacity domains. This evaluation identified improvements that are now being implemented.

## Introduction

Engaging in health research is important for identifying and prioritising the health needs of a population. The lack of health research capacity in low- and middle-income countries (LMICs) hinders their ability to respond to the needs of their communities.^1^ This is especially true for primary care research in LMICs.^2^ Health research capacity building, or strengthening, is defined by Lansang et al. as the ‘ongoing process of empowering individuals, institutions, organizations and nations to define and prioritise problems systematically, develop and scientifically evaluate appropriate solutions, and share and apply the knowledge generated’.^1,3^ Family medicine postgraduate training programmes in sub-Saharan Africa have recognised the importance of research training as a necessary part of their curricula. The completion of a scholarly project is a common requirement for graduation from these programmes.^4,5,6^ Physicians who are equipped with skills to generate hypotheses, design methods to test them and analyse collected data can enact change in their communities.^7^ Requiring family medicine trainees to conduct research is a promising strategy to increase needed primary care research capacity.^8^ The completion of the research requirement, however, can also create significant bottlenecks in training and even become a negative experience for family medicine trainees.^5,9^ Such difficulties occur if trainees are not provided with sufficient research training and mentorship to complete the required project. Nevertheless, according to findings from a meeting report by Yakubu et al., it is possible to move family medicine research engagement beyond a ‘mere obligation’ required for graduation by making the experience enjoyable, developing internal motivation of the trainees and providing sufficient mentorship.^5^

There is a growing literature recognising the crucial role that mentorship plays in research capacity building.^10^ Specifically, peer and ‘near-peer’ mentorship can enhance learning and motivation, providing an avenue for open communication outside of the typical medical education hierarchy. ‘Near-peer’ mentorship occurs between individuals who are typically one level of training different from one another, for example, between undergraduate and graduate students.^11^ There have been evaluations of peer and near-peer mentorship programmes for pharmacy student researchers, geriatric post-doctoral fellows and oncology clinician researchers. Common amongst these studies has been the success of the peer and near-peer model to fill a resource gap in available research mentorship for trainees.^12,13,14^ The authors were unable to identify any literature evaluating the use of peer or near-peer mentors within family medicine postgraduate research training in sub-Saharan Africa.

Typical measures to assess the success of research capacity building programmes include a number of peer-reviewed publications, presentations at conferences, grant applications and attainment of higher degrees; however, these are challenging to obtain in low-resource and early-career settings. In addition, these metrics fail to capture a primary goal of health research capacity building, which is to impact the health of and service delivery to the local community.^15,16^ Cooke’s framework for evaluating research capacity building focuses on ‘process’ domains that are relevant to novice researchers and can be measured more proximally. It also focuses on ‘outcome’ domains that consider the community health impact of research, which are especially relevant to family medicine. These domains include: (1) building skills and confidence, (2) ensuring that research is ‘close to practice’, (3) supporting linkages and collaborations, (4) developing appropriate dissemination, (5) building sustainability and continuity and (6) investing in infrastructure.^16^ We, therefore, utilise Cooke’s framework to evaluate the family medicine training programme’s efforts to build research capacity through its research curriculum and a novel peer mentorship programme. This article describes the research curriculum, the peer mentorship programme and its evaluation.

## Methods

We conducted a longitudinal mixed-methods evaluation of the research training component of the Lesotho-Boston Health Alliance (LeBoHA) Family Medicine Specialty Training Programme (FMSTP) curriculum.^17^ The curriculum uses peer research mentors from the United States (US) to support FMSTP postgraduate trainees remotely as they complete a required research project. The primary goal of the curriculum and mentorship programme is to build research capacity amongst the FMSTP trainees. The specific objectives of this evaluation were to: (1) understand the impact of these efforts on trainee’s research capacity, (2) evaluate the use of peer mentorship to support FMSTP research training and (3) generate insights to improve the quality of research training and mentorship in Lesotho and elsewhere.

### Setting

Lesotho is a small landlocked country within South Africa, which faces the highest rates of human immunodeficiency virus and tuberculosis in the world. Approximately half of Lesotho’s two million people, called Basotho, live on less than 1.90 USD per day.^18^ Lesotho does not have a medical school and the FMSTP is the first and only accredited postgraduate medical education programme in Lesotho. The FMSTP is an academic partnership with the Lesotho Ministry of Health and the LeBoHA.^19^ Over the four-year programme, trainees are educated in clinical family medicine, public health and district health management, including a required scholarly research project in a primary care topic relevant to their community.

### Authors’ relationship to topic

Grounding our methods in reflexivity, the following is a brief explanation of the authors’ role in both the implementation and evaluation of the FMSTP research curriculum and mentorship programme.^20^ The first author, C.M., is a family physician and currently the research director of the FMSTP. She developed and implemented the peer mentorship structure, including recruitment and matching of peers, and serves as peer mentor herself. The fourth author, B.J., is a family physician and the director of LeBoHA. He and C.M. are responsible for teaching the majority of the FMSTP research curriculum. B.J. also provides senior faculty-level research mentorship to all FMSTP trainees. Second author, K.R., joined the evaluation as part of her Master of Public Health training. K.R. became a peer mentor to one of the current FMSTP trainees in April 2019. Third author, S.M., is the first family medicine graduate of the FMSTP and is now its director. He provides faculty research supervision to the trainees. Last author, C.B., is a global mental health researcher who has visited the FMSTP programme, but does not play a direct role in the FMSTP curriculum nor mentorship. Co-authors S.M. and B.J. participated as faculty members in the semi-structured interviews that were included in this analysis. None of the other co-authors participated as subjects in the evaluation.

### Research curriculum

**FIGURE 1:**
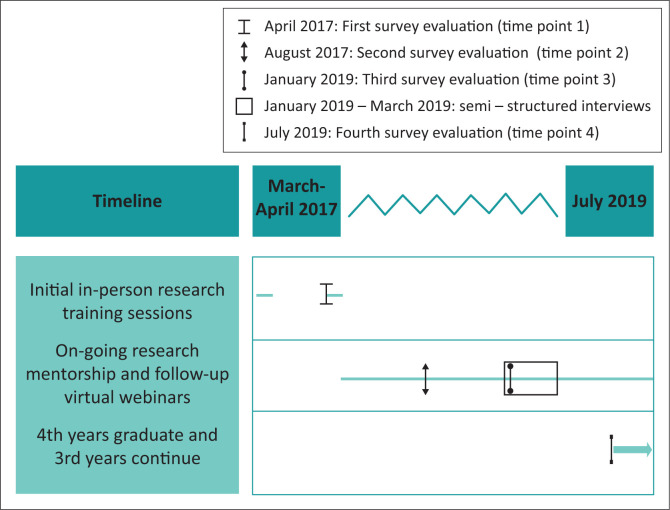
Timeline of Family Medicine Specialty Training Programme 2017–2019 research training and mentorship and its longitudinal, mixed-methods evaluation.

The overall FMSTP curriculum is taught via monthly in-person week-long ‘contact sessions’, coupled with intermittent remote training and supervision visits to trainees in the district hospitals where they are employed. In recent years, FMSTP training increasingly involves blended learning strategies that use online resources and instruction to complement face-to-face instruction.^21^ The curriculum includes separate components on community-oriented primary care (COPC), quality improvement and research. The four second-year and four third-year trainees started the research curriculum together during the March 2017 contact session. This session introduced basic research theory and design fundamentals. Participants learnt to create their own problem statement, research question, select a methodology and to search online databases. Following this, each trainee had in-person supervision visits focused on literature review, stakeholder engagement and advancing their individual proposals. The next contact session included a dedicated session on research ethics and rolled out the research mentorship structure (see below). All remaining sessions were taught remotely using one-hour virtual webinars targeted to occur before specific next steps in the research process. For example, a remote Institutional Review Board (IRB) protocol development webinar occurred before the trainees started writing their research proposals. A research data management webinar was held prior to the majority of trainees started their data collection. Additional research training occurred via feedback and discussion between trainees and mentors during the process of creating and implementing their research protocols. See Figure 1 for an overview of the curriculum timeline.

### Research mentorship

To support the research training curriculum, a peer research mentorship programme, which paired the US and FMSTP post-graduate trainees, was developed and implemented in April 2017. Peers were recruited via an email to the Boston University Family Medicine residency listserv and via personal invitation. Although there was no formal requirement for level of research expertise to become a peer mentor, all had previous experience in conducting research. The mentorship interactions occurred primarily via email and social media platforms, such as WhatsApp. The US peer mentor assisted the Lesotho trainee throughout the research process. This included brainstorming research ideas, identifying relevant literature, editing drafts of proposals, presentations, posters and manuscripts and generally learning research skills together. Peers were expected to communicate regularly with trainees, at least once every six weeks. Trainees and peers had the support of senior faculty research mentorship, provided by B.J., who was available for questions and feedback. All trainees were additionally assigned a Lesotho-based faculty research supervisor. Lesotho faculty have limited prior research experience, and thus, the role of these supervisors was primarily to assist with on-the-ground troubleshooting and support trainee’s progress within the context of the FMSTP training programme.

### Evaluation participant recruitment

This evaluation firstly included all eight FMSTP trainees who were engaged in the research training curriculum and all Lesotho-based FMSTP faculty. As the evaluation continued, all FMSTP administrators, Boston-based FMSTP faculty and all US peer mentors, apart from author C.M., were also invited to participate. This resulted in a convenience sample of participants from each of these categories who were available and willing to offer feedback at each evaluation time point over a two-year period.

### Tool development

The evaluations were conducted using three main tools. Firstly, the FMSTP programme already uses a trainee self-evaluation tool on which the trainees rank their confidence in various curricular domains via a simple five-point Likert, with a 1 representing ‘not confident’ and a 5 representing ‘very confident’. This was adapted to assess confidence in the seven key research skills the curriculum was designed to teach: (1) choose a research question, (2) conduct literature review, (3) design study, (4) collect data, (5) analyse data, (6) write results and (7) present research. The trainee self-evaluation tool results were not anonymous, as they were available to faculty and administrators of the programme. Secondly, a general FMSTP programme feedback form was adapted into a simple short-answer survey, asking the respondent to comment on: (1) their overall impression of the peer mentorship programme, (2) programme strengths, (3) current or anticipated challenges and (4) ideas for improvement. This programme feedback tool was anonymous. Both tools were piloted with a trainee, who provided feedback on question clarity and understandability prior to administration (see Appendices 1 and 2). Thirdly, evaluators developed a question guide to facilitate semi-structured interviews. These guides were designed to generate information that would expand upon and triangulate with survey data. The guides specifically sought to (1) understand the impact of the FMSTP research training and mentorship approach on trainee research capacity, (2) explore the experience of peer research mentorship for the trainees, peers, faculty and programme administrators and (3) identify future directions for improving quality of research training and mentorship (see Appendix 3).

### Data collection

This two-year evaluation invited participation from 20 individuals in total, including trainees, faculty, administrators and peers, with variable participation at each time point. Trainee self-evaluation surveys were collected from all trainees at three time points: April 2017 (pre-programme, T1), August 2017 (early midpoint, T2) and January 2019 (late midpoint, T3). Post-programme (T4) survey data were collected in July 2019 from the graduates only. Surveys were administered in paper format, while C.M. was in-country during the T1 and T2 evaluations. Subsequent surveys were then administered via Qualtrics online survey software (Qualtrics, Provo, UT).^22^ K.R. conducted the majority of interviews. These were conducted both in-person and via web-conferencing software (Zoom) during January–March 2019. See Figure 1 for evaluation time points. Those who could not participate in interviews were given the option to email responses to the interview questions. Demographics were collected via programme logs, verbally at the time of interview or via email.

### Quantitative data management and analysis

Paper surveys were entered into Microsoft Excel and combined with exported Qualtrics survey data. Descriptive statistics of participant demographic data, such as means, medians, standard deviations and proportions, were calculated using RStudio (Version 1.2.1335).^23^ Programme faculty and administrator demographics were analysed and are reported in aggregate to help protect participant anonymity. Likert scores across all seven measured domains were averaged to create a single ‘survey-scale’ research confidence score for each trainee.^24^ Changes in research confidence scores were analysed and compared using medians and ranges. Given T4 data were available only for the graduates, these four individuals were also analysed independently.

The non-parametric Friedman test was used to assess significance of changes in Likert-scale confidence scores in all seven research domains for all trainees combined and for graduates alone, across their respective time points. The sign test was then used to assess if the change was positive or negative when comparing T1 to T3 for all trainees and comparing T1 to T4 for graduates.

### Qualitative data management and analysis

All interviews were audio-recorded, transcribed and coded with the support of NVivo (Version 12.6.0).^25^ We used a semi-verbatim transcription style that allowed for removal of false starts and interviewer prompts to improve clarity, but preserved all content of text. All written survey data were entered into a table and also edited for spelling and punctuation. Interview transcripts and written data were analysed through the use of thematic analysis using a mixed inductive–deductive approach.^26^ Analysis was both data-driven and theory-driven, using questions from the interview guide as well as Cooke’s framework to develop an initial *a priori* codebook.^16^ This framework was selected prior to development of the codebook as a means of ensuring that our analysis included a review of each core domain of research capacity building.

Both K.R. and C.M., initially familiarised themselves with the data through a quality assurance phase, in which all transcripts were checked against the original audio. K.R. and C.M. conducted open coding of two interviews of different participant types and the resultant concepts were combined with the *a priori* codes to create an initial codebook. This codebook was then used by both researchers to code four additional interviews, with review and collaborative modification of the codebook after each. The codebook was shared with S.M. in Lesotho for feedback. A fifth interview and a randomly selected set of written interview responses were coded with this pre-final codebook and given no additional concepts from the data were identified, and the codebook was finalised. After finalising the codebook, one interview was independently coded and inter-coder agreement was calculated to be 97.3%. This final codebook was then used by C.M. and K.R. to independently code all subsequent interviews and written responses, and to recode the initial interviews that were used to develop the codebook. After all data were coded in NVivo, both K.R. and C.M. reviewed coded segments independently to identify themes via relationships between codes and to Cooke’s framework domains. These themes were then reviewed collaboratively together and in a meeting with S.M. to both finalise the analysis approach and begin to define the core themes to explore. K.R. and C.M. then collaboratively mapped themes onto Cooke’s framework and iteratively defined, and then named those that fell outside of the framework but were prominent in the collected data.

Memoing was used extensively throughout the transcription, quality assurance, familiarisation, coding and analysis process to encourage reflexivity, especially regarding the ways in which the evaluators’ role in the delivery of the FMSTP curriculum and mentorship may influence its evaluation.^27^ In addition, we made use of member checking of our preliminary analysis to provide all participants the opportunity to comment on the appropriateness of data interpretation and representation of the programme.^28^ A table containing preliminary findings and exemplary quotes was shared with all invited participants and a three-week period was allowed for comments. During this period, 11 participants responded with feedback and all approved the results.

Finally, the analysis methods above describe primarily the summative data analysis process. In addition, shortly after each survey evaluation time point, self-evaluation results were shared with the FMSTP faculty, trainees and their research mentors to allow for discussion and action on the identified challenges. This iterative quality improvement approach allowed for real-time adjustments throughout the programme.

### Ethical consideration

Ethical approval was obtained from the Boston University Medical Campus IRB and the Lesotho Ministry of Health Research Ethics Committee. All written surveys included a research opt-out clause and verbal informed consent was obtained for each interview participant. Verbal consent was chosen to allow for ease of conducting both in-person and web-conference interviews and because of the low-risk nature of the study. Ethical clearance numbers: Lesotho Ministry of Health Research Ethics Committee Number: ID89-2017. BU IRB Number: H-36847

## Results

### Characteristics of our sample

The demographics of all 20 invited participants are summarised in Table 1. Invited trainees had a 100% response rate at all evaluation time points. Six of the eight trainees (75%) participated in semi-structured interviews. Across both class years, half of the trainees were female and their average age was 36.4 years old. All trainees were Basotho. Faculty participation varied over the two-year period because of the retirement and hiring of new faculty. At least two Lesotho-based faculty members completed an anonymous written programme evaluation at each time point. Four faculty participated in semi-structured interviews and one provided written interview answers via email. Three administrators were invited to participate in the programme evaluations at the T3 and T4 time points and one administrator completed an interview. Programme faculty and administrators were mostly male and included Basotho, American and German nationalities. All three peers invited to participate in the evaluation are female and are from the United States. Of these, only one peer provided an emailed interview response and two provided survey responses.

**TABLE 1:** Demographics of invited Family Medicine Specialty Training Programme research training and peer mentorship evaluation participants (*n* = 20).

Characteristics	*n*	%	Mean	s.d.
**Family medicine trainees (*n* = 8)**				
Age, years	-	-	36.4	4.6
*Gender*	-	-	-	-
Female	4	50.0	-	-
Male	4	50.0	-	-
*Nationality*				
Basotho	8	100.0	-	-
**Programme faculty and administrators (*n* = 9)**				
Age, years	-	-	46.6	15
*Gender*				
Female	3	33.3	-	-
Male	6	66.7	-	-
*Nationality*				
Basotho	4	44.4	-	-
German	1	11.1	-	-
United States of America	4	44.4	-	-
**Peer mentors (*n* = 3)**				
Age, years	-	-	34.3	5.1
*Gender*				
Female	3	100.0	-	-
*Nationality*				
United States of America	3	100.0	-	-

s.d., standard deviation.

### Quantitative results

When evaluating trends in overall research confidence scores amongst all trainees (*n* = 8), we saw an increase in the median score of 1.7 (range 1.0–2.9) at T1 to a median of 2.6 (range 2.1–3.1) at T2. There was no further increase at T3, however, with the median score remaining the same at 2.6 points on a five-point scale. A Friedman test of differences amongst repeated measures conducted on the raw Likert scores for all trainees across these three time points was statistically significant (*X*^2^ = 37.33, *p* < 0.001). A two-sided sign test conducted on trainees’ T1 compared with T3 raw Likert scores showed a positive median increase of one point.

**FIGURE 2:**
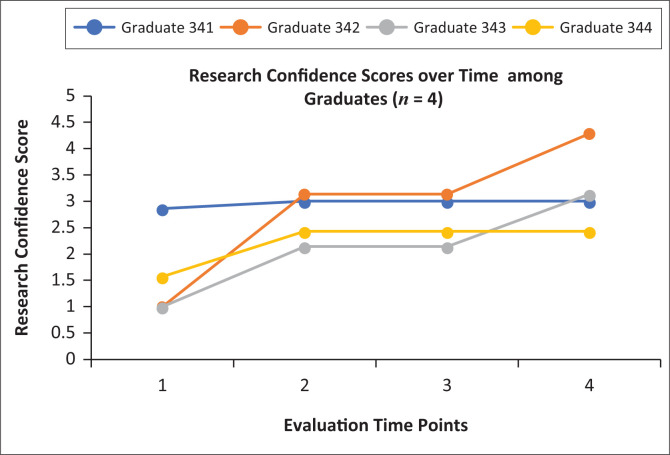
Research confidence scores among Family Medicine Speciality Training Programme graduates increased over the four evaluation time points. These scores were calculated by averaging the Likert ranks across all measured domains at each time point, with one representing ‘not confident’ and five representing ‘very confident’. Domains included: (1) choose a research question, (2) conduct literature review, (3) design study, (4) collect data, (5) analyse data, (6) write results and (7) present research. Graduates’ names have been replaced by a numeric code starting with a three, which indicates that they started the research curriculum in the third year of the programme.

Figure 2 shows the change in graduates’ (*n* = 4) research confidence scores over all four time points. As an aggregate, we saw a 1.8 point rise in median research confidence scores between T1 (1.3, range 1.0–2.9) and T4 (3.1, range 2.4–4.3), although gains varied by individual graduate. A Friedman test on the raw Likert scores of the graduates across all four time points was also statistically significant (*X*^2^ = 34.48, *p* < 0.001). A two-sided sign test conducted on graduates’ T1 as compared with T4 raw Likert scores showed a positive median increase of two points.

### Qualitative results

**FIGURE 3:**
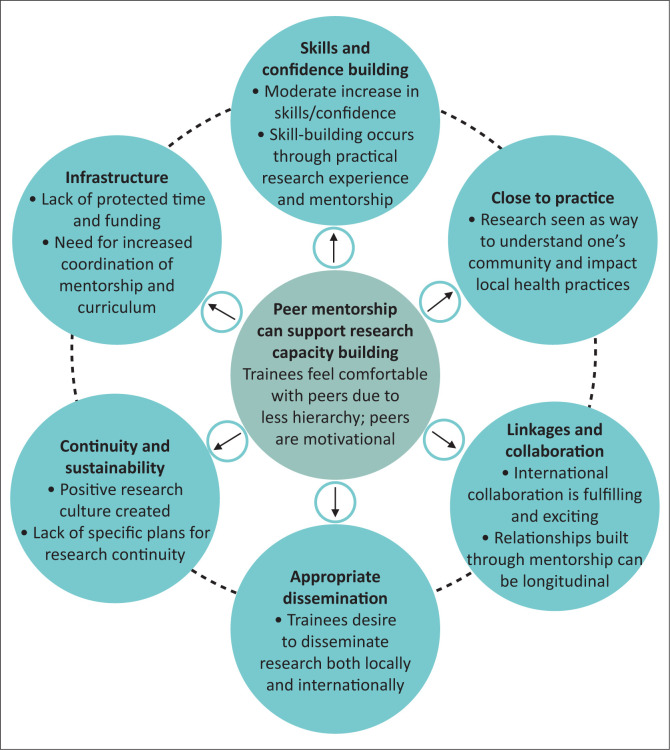
Key findings of our qualitative evaluation of the Family Medicine Specialty Training Programme research training curriculum supported by peer mentorship are summarised here. The findings depicted in the outer circles are organised by the six domains of Cooke’s framework.^16^ The findings depicted in the centre circle are specific to the theme of peer research mentorship.

Ten semi-structured interviews, two written interview answers and all 36 open-ended survey responses were analysed. Results were organised by the six domains of Cooke’s framework. Where appropriate, distinction is made between the individual, organisational and supra-organisational levels at which these domains operate. We share additional findings specific to peer mentorship that emerged from the data. Representative quotes are identified using the numerical code of the participant. Trainees who began the research curriculum of the FMSTP as a second year have codes starting with two, whilst trainees who began the research curriculum as a third year have codes starting with three. See Figure 3 for a summary of results.

### Skills and confidence building

Trainees built research skills and confidence primarily through the practical experience of conducting a mentored research project. This intentional ‘learning-by-doing’ approach placed research mentorship in a central role to support trainees’ research skill development:

‘Unless you’re using that information (…) it doesn’t mean anything, you have to apply it to a project and have a mentor working with you on the project for that to make sense.’ (Faculty 191, Male)

Although research skills increased, confidence in using those skills, for example, to go on and be a peer mentor themselves, was limited for most trainees. Just one trainee expressed that he or she would feel confident being a peer mentor, but others were hesitant, not feeling ready yet:

‘From the experience that I have now, I think I can be able to help people and show them how to find information.’ (Trainee 342, Female)

‘At the moment I can’t be a mentor because I still need to be mentored.’ (Trainee 343, Female)

In addition, a highly applied research training curriculum can also result in trainees developing skills that relate to their own project but lack a broader familiarly with other research types. This resulted in many trainees feeling limited in their self-efficacy of overall research abilities:

‘I can’t appreciate lots of things with research. I just know what I’m doing.’ (Trainee 343, Female)

### Close to practice

Trainees consistently made statements demonstrating their understanding that research has the potential to greatly impact local practice and that community-based research is an important vehicle for physicians to learn about their community:

‘Up until you have done the research, met the community, got the information from the community, it’s up until then that you get the reality of what’s going on (…) this has just opened my eyes.’ (Trainee 341, Male)

To ensure that research does remain ‘close to practice’ research topic selection and questions must be defined locally. Trainee research should flow from the COPC portion of the FMSTP curriculum and must be ‘operational’, designed to solve a problem that exists in the community or institution:

‘The problems and the project come from them, not any external suggestion or anything from well-meaning peer mentors.’ (Faculty 112, Female)

‘Focusing on operational research that flows from the COPC is going to make it more useful (…) [*it will*] reinforce COPC which is one of the core aspects of family medicine in this region.’ (Administrator 142, Female)

### Linkages and collaboration

The peer mentorship itself was an important collaborative experience that was built into the FMSTP approach to research training. Having peers come from abroad provided an additional ‘excitement’ factor:

‘It’s so exciting (…) to have those kind of relationships (…) ‘I have someone that I can talk to from abroad.’ (Administrator 142, Female)

The programme also included an opportunity for trainees to present their research at the World Organization for Family Medicine’s (WONCA) Africa Regional Conference. This provided an opportunity for regional networking and idea exchange:

‘It was fulfilling to exchange ideas with people from different cultural backgrounds.’ (Trainee 231, Male)

The findings highlight the importance of research mentorship to foster linkages that can be sustained throughout one’s career:

‘There’s an ongoing relationship after that, ideally. And not just with the mentor, but with other people who are doing similar things. You have a cohort of people who are then your peers in that research area (…) they have a long, lifetime often, relationship around the work they are doing.’ (Faculty 191, Male)

A number of the trainees’ research projects engaged nurses to support with data collection, for example, to co-facilitate a focus group on barriers to cervical cancer screening. There was limited other discussion in the interviews, however, regarding local stakeholder engagement, inter-professional research collaborations or linkages with policy makers.

### Appropriate dissemination

Trainees were asked on the written surveys about their goals for their research projects and many expressed a desire to disseminate their results both within Lesotho and internationally:

‘Want it to be published nationally and globally.’ (Trainee 233, Female)

‘To present it at [*a*] symposium, nationally and internationally.’ (Trainee 231, Male)

Beyond these traditional means of dissemination via presentations and publications, trainees did also express goals that acknowledge research’s potential to impact local health practices:

‘Improve cervical cancer screening in my district because it really is preventable.’ (Trainee 341, Male)

Lacking, however, were more specific plans for strategic local dissemination that would lead to changes in their clinical environments or health policy.

### Continuity and sustainability

Trainees expressed an appreciation for the continuity of the mentorship relationship with their peers throughout the research training experience:

‘Very helpful and [*they*] guide us through every step.’ (Trainee, anonymous evaluation)

Participants spoke about the importance of research for family medicine, seeing it as a way to improve practices and overall systems, and reveal what is going on in their communities. This, in addition to a number of trainees explicitly stating their desire to continue engaging in research, indicates that a positive research culture was fostered through the programme:

‘There’s so much more to know about my community.’ (Trainee 341, Male)

‘So family medicine is creating this culture of research in us, [*a*] culture of living and doing research.’ (Trainee 232, Male)

Apart from the idea of involving the graduates as local near-peer research mentors, no other specific plans, funding or structure for continued involvement in primary care research beyond trainees’ FMSTP research project were discussed.

### Infrastructure

A number of infrastructure-related challenges were noted at all levels of Cooke’s framework. Firstly, trainees did not have protected time to dedicate to their research projects and struggled with time and overall project management:

‘We are supposed to do the work on our own time, so I think that one was a bit difficult because sometimes we are overrun by the other jobs, so we ended up giving minimum time to research.’ (Trainee 342, Female)

Also, at the individual level, a lack of funding to support the costs of conducting the research was noted:

‘They talk about issues of resources, even if it seems small, as being difficult. Like making copies, having toner, having pens, having adequate space and time to complete everything.’ (Faculty 132, Male)

At the organisation level, there is a need for research capacity building among the Lesotho-based FMSTP faculty. This will improve their provision of academic supervision for the trainees and remove their dependence on Boston for this support:

‘Let us also make sure that our faculty members get the capacity they need, so that when there is no such programme, they can be able to play that part.’ (Administrator 142, Female)

Additionally, there was a lack of comprehensive coordination of the research curriculum and mentorship programme. Many felt pressure in relation to the programme timeline and expressed that better organisation and coordination would have helped them feel more prepared:

‘I wish that the objectives could be very clear (…) so that everybody knows from the beginning what they are expected to do. And then we don’t get surprises in the middle of the programme.’ (Trainee 341, Male)

The research mentorship occurred largely virtually, which was described as a flexible and effective way to have questions answered promptly:

‘Even when she left Lesotho to the States, she kept communication going on because she opened up a WhatsApp group (…) So, in a way, she’s always in Lesotho. Every time I say anything, [*she*] will just pop up and answer me, at any point, at any time.’ (Trainee 341, Male)

In contrast, some trainees also expressed that virtual mentorship has limitations, and sometimes, in-person support would be preferred. National infrastructure challenges of having a poor network and the cost of data in Lesotho were also discussed:

‘We have just recently begun to have phone contact for each other, as it was sometimes a bit difficult with emails alone.’ (Trainee, anonymous evaluation)

‘Communication can be difficult over internet, data in Lesotho is expensive, and the network is bad many times.’ (Faculty, anonymous evaluation)

In Lesotho, there is one national IRB through which all human subjects research protocols are evaluated. The approval process was lengthy for all trainees and resulted in delays in starting their projects. Additionally, there is only limited locally produced data to inform research studies:

‘The same studies might have been done abroad, or somewhere before, but the local data is not there.’ (Trainee 232, Male)

### Peer mentorship

In addition to the themes found in relation to Cooke’s framework, another major theme relates to the use of peer mentorship as a unique approach to support research capacity building. Faculty, administrators and trainees alike noted the unique benefits to having a peer as part of the research mentorship process. Because of less hierarchy in a peer relationship, trainees feel open and are more comfortable asking questions. This was contrasted with supervisors, whom trainees often noted that they feel less comfortable with. Peers use informal communication methods that make them easily accessible and many times were referenced as a ‘friend’:

‘It’s easier to talk with [*a*] peer mentor than the supervisors. And they are informally available, on WhatsApp or Facebook.’ (Trainee 343, Female)

‘Lowers barriers to ask for assistance and it promotes motivation.’ (Faculty 122, Male)

‘My understanding is that a peer is someone whom we can be friends with (…) and is someone that I can freely learn from.’ (Administrator 142, Female)

As peers have been through the training process recently and are themselves managing similar challenges, they are relatable. These peer relationships were described as motivating and good at providing ‘moral support’. A limitation to peer mentorship is that they may not have the time to be as responsive as desired because they are themselves in-training or busy early-career physicians. Additionally, there were concerns about the role of the peer and whenever they have sufficient expertise to provide valuable research mentorship:

‘She tries by all means to be quick at response, but certain times it might not be as quick as expected.’ (Trainee 232, Male)

‘Some US residents are not equipped or available enough to be good peer mentors.’ (Faculty, anonymous evaluation)

‘I wish I had more research knowledge to bring to the table.’ (Peer mentor 172, Female)

Throughout the interviews, respondents enumerated specific characteristics of good peer mentors. These included being: (1) good communicators, including being adept listeners and prompt with responses, (2) dedicated and committed, (3) friendly and non-judgemental, (4) good at time management, and specifically being able to model and teach time management, (5) experienced in research and (6) familiar with local context of mentee.

#### Triangulation of quantitative and qualitative results

Moderate increases in research confidence among trainees were seen in both survey results and via the interviews. After completion of the programme, the four graduates’ median research confidence score of 3.1 represented a significant improvement from their pre-programme score; however, it still only reached moderate levels of confidence on the 5-point Likert scale. The interviews allow us to unpack these scores. Trainees expressed that while they do feel ‘a little’ confident, most do not yet feel sufficiently confident to do a research project independently, nor mentor another without additional support:

‘I feel a little confident. We did the protocol (…) and I feel okay, it’s doable, I can do it, maybe.’ (Trainee 343, Female)

‘Continue to get better at it until I am able to mentor someone else.’ (Trainee, anonymous evaluation)

## Discussion

The evaluation demonstrated that FMSTP research curriculum and peer mentorship programme were successful in positively impacting a number of Cooke’s research capacity building domains.^16^ Firstly, research skills and confidence increased moderately among FMSTP trainees over the two-year evaluation period. Graduate’s research confidence scores increased as a group, although individual graduates varied. Specifically, graduate 342 started with low confidence that increased substantially, while graduate 341 started with relatively high confidence and made minimal gains. All graduates completed their research projects within their four-year training programme and published research articles in *Lesotho Medical Association Journal*.^29,30,31,32^ One graduate presented her final research results at the *South African Academy of Family Physicians 22nd National Family Practitioners Congress* in August 2019.

The programme was also successful in creating research experiences that were grounded in the trainee’s clinical practice and enhancing of the overall ‘culture of research’ within the FMSTP, which is promising for sustainability. As was noted by Cooke, these latter two successes feed one another; the more that the trainees engage in ‘useful’ and locally relevant that is ‘close to practice’, the more they value research engagement.^16^ According to Mash et al.,^4^ the development of a research culture is ‘essential’ to advance African primary care research capacity building.

Other key findings of the evaluation included the identification of specific benefits of having peers as research mentors. Trainees feel comfortable with peers, who are seen as friends and are readily available via informal communication methods. Virtual delivery of peer mentorship is possible and itself has the benefit of being highly flexible, although it is limited by Internet connectivity and cost. Teaching research via an applied, learning-by-doing approach is valuable. However, it must be balanced with formal instruction on research theory and opportunities to learn about other trainees’ research projects to ensure exposure to a variety of research methodologies. The evaluation identified that more work needs to be performed within the domains of linkages and collaboration, specifically with regard to community engagement, appropriate dissemination and continuity and sustainability. Another key finding of the evaluation was the identification of a number of research infrastructure-related gaps within the FMSTP. These included insufficient protected time, lack of funding for research-related costs and need for clearer organisation of the research curriculum and mentorship structure.

Many of our findings regarding peer research mentorship are similar to those found by Rukundo et al.^11^ in their study of near-peer mentorship of medical undergraduates by master’s students. Peers are able to increase the workforce within institutions that lack sufficient mentorship capacity and can bridge gaps between senior lecturers and learners. In both studies, participants mentioned feeling more ‘free’ to engage with and ask questions of peers than senior lecturers. Cole et al.^10^ focus on the need to create ‘safe spaces’ for mentorship to be able to thrive. They also highlight how focusing on co-learning can promote the development of mentorship ‘across hierarchies’. Our study supports this concept that the creation of a safe, non-hierarchal learning environment is a key benefit of peer mentorship.^10,11^

Lescano et al.^33^ highlight a nuance to our study’s finding regarding peer mentorship’s ability to circumvent the hierarchy typically found within the medical training. They argue that mentorship culture in high-income countries (HICs) tends to be more horizontal, whereas in LMIC, it tends to be more strictly hierarchical. Thus, although it is tempting to attribute our findings exclusively to the fact that the mentor is a peer, it may also be because of our peer mentors coming from the United States, an HIC. These peers may have brought their own cultural norms and approach towards mentorship that could, in turn, influence the lack of hierarchy, just as much as the peer aspect. Characteristics of a ‘good’ peer mentor that were identified in our study, such as being committed, available, experienced in research and a good listener, mirror those found in recently published LMIC research mentorship competencies.^33,34^

The described FMSTP research training curriculum and mentorship structure lacked sufficient organisation, causing the trainees to feel uncertain in terms of expectations. Literature evaluating family medicine resident scholarship has specifically identified uncertainty as a key barrier to research engagement.^35^ The infrastructure gaps we identified, including lack of protected time and research funding, are common within family medicine residency training programmes worldwide.^36^ A study of family physicians in Kenya identify these same barriers as important limitations to continuing research engagement beyond residency training, despite ongoing interest and recognition of research as important to family medicine practice.^37^

Using the domains of Cooke’s framework to organise our analysis had a number of benefits; however, there were also some limitations to its use. Some gaps that were noted in specific domains are likely partially attributable outside factors, such as the timing of the interviews and the content of the interview guide. The interviews took place mid-way through the research curriculum. This timing may be the primary reason the interviews failed to capture specific plans for local dissemination of trainees’ research results. Similarly, we have only limited information about continuity and sustainability of the programme given this timing. The interview guides were not explicitly developed to capture all of Cooke’s framework domains, and thus, gaps in areas such as linkages and collaboration may be because of the failure of asking specifically for this information.

Other limitations include that this was an evaluation of a single training programme, and thus, our case study may not be generalisable to family medicine programmes in other contexts. Our small sample size and use of averages to report Likert-scale data limits interpretation of changes in research confidence. As the authors were involved in both implementation and evaluation of the programme, the study may be subject to researcher bias. Response bias may also limit the findings, which were heavily drawn from the semi-structured interviews. Two of the eight registrars were not able to be interviewed, and only one peer mentor provided written responses to the interview questions, but no full interviews were conducted with peers. A final limitation was that the evaluation did not systematically capture the level of engagement of each trainee with the research curriculum or with their peer mentorship. This limits the interpretations of findings because we cannot accurately report on the dose of the intervention received by each trainee.

These limitations are mitigated, however, by the use of triangulation during this longitudinal mixed-methods evaluation. We elicited perspectives from trainees, faculty, administrators and peers both via anonymous written feedback and via in-depth interviews. Other methodological strengths included rigorous quality assurance of all transcriptions, an iterative process of developing the codebook with the inputs of three authors, consistent use of memoing focusing on reflexivity and using member checking to ensure the validity of our findings.

This evaluation was successful in its objective of identifying ways to improve quality of the FMSTP research training and mentorship approach. In November 2019, C.M. presented the findings of this study to FMSTP faculty. This resulted in several changes, such as a more clearly structured research curriculum, including explicit learning outcomes that were added to the FMSTP training portfolio. The overall curriculum was reorganised to ensure that trainees do a COPC needs assessment before initiating their research projects. Research-in-progress meetings were added to quarterly contact sessions and trainees will now have approximately eight afternoons of protected time for research during specific clinical rotations each year. A revised structure for research mentorship and supervision was implemented, including the use of local near-peer research mentors, who are the four recently graduated family medicine specialists.

Future studies are needed to assess the value of the changes made within the FMSTP based on this evaluation. In addition, although our study supports the use of peer and distance mentorship, further work is needed to understand how these strategies may be useful in other contexts.

## Conclusion

Equipping family physicians with the capacity to ask and answer important research questions in their communities holds the promise of improving primary care health delivery, especially in a resource-limited context such as Lesotho. This evaluation of the FMSTP research curriculum and peer mentorship programme directly resulted in a number of specific improvements that are being implemented, including better organisation of the research training curriculum, the addition of protected time for research and the use of recent graduates as near-peer research mentors. Our example of using Cooke’s framework to evaluate our programme may guide further research in this crucial area of research capacity building for family physicians in LMICs.

## Appendix 1

### The Family Medicine Specialty Training Programme registrar self-evaluation of research skill confidence

This form is intended to help us understand where you perceive your research skills to be, understand more about your research-related goals and how we can help you reach them.

Think about your research skills right now, how confident are you in your ability to:

1. **Choose a research question**
  **Table:**
  Not confident		Somewhat Confident		Very Confident
  1	2	3	4	5
2. **Conduct a literature review**
  **Table:**
  Not confident		Somewhat Confident		Very Confident
  1	2	3	4	5
3. **Design a research study**
  **Table:**
  Not confident		Somewhat Confident		Very Confident
  1	2	3	4	5
4. **Collect and manage research data**
  **Table:**
  Not confident		Somewhat Confident		Very Confident
  1	2	3	4	5
5. **Analyze the collected data**
  **Table:**
  Not confident		Somewhat Confident		Very Confident
  1	2	3	4	5
6. **Write a dissertation or paper for publication**
  **Table:**
  Not confident		Somewhat Confident		Very Confident
  1	2	3	4	5
7. **Present your research in a Poster session or PowerPoint presentation**
  **Table:**
  Not confident		Somewhat Confident		Very Confident
  1	2	3	4	5

What goals to you have for your research? _______________________________________________________________________________

____________________________________________________________________________________________________________________

What are you struggling with, in regard to your research?___________________________________________________________________

____________________________________________________________________________________________________________________

How can we help you reach those goals? ________________________________________________________________________________

____________________________________________________________________________________________________________________

## Appendix 2

### The Family Medicine Specialty Training Programme Peer Research Mentor* Programme feedback surveys for trainees, peer mentors, faculty and administrators.

***Note: The term ‘peer partner’ was used in place of the word ‘peer mentor’ in these tools**

#### 2.1. Version for trainees (referred to below as ‘registrars’)

This form is intended to help us understand how the peer research partnership program within the FMSTP is working and how it can be improved.

What is your overall impression of having a peer research partner work with you on your research project?

____________________________________________________________________________________________________________________

____________________________________________________________________________________________________________________

Areas of strength of peer research partnership:

____________________________________________________________________________________________________________________

____________________________________________________________________________________________________________________

Areas of peer research partnership to be improved or challenges:

____________________________________________________________________________________________________________________

____________________________________________________________________________________________________________________

Ideas for improvement of peer research partnership:

____________________________________________________________________________________________________________________

____________________________________________________________________________________________________________________

#### 2.2. Version for peer mentors

This form is intended to help us understand how the peer research partnership program within the FMSTP is working and how it can be improved.

In what ways have you interacted with your registrar research partner (i.e. email communication, text messages, voice call, video call, in person meeting)? Please list all means you have used to-date and if applicable what application you used, such as: Whatsapp, Skype, Google Hangouts, GoToMeeting, etc: __________________________________________________________________________________________

____________________________________________________________________________________________________________________

Approximately how many interactions have you had with your registrar research partner to-date, with one interaction defined as: one email thread, one text exchange, one call, etc? Please choose the single best answer:

[ ] 0 interactions

[ ] 1–3 interactions

[ ] 4–6 interactions

[ ] 7 or more interactions

What is your overall impression of having peer research partners work with registrars on their research projects?

____________________________________________________________________________________________________________________

____________________________________________________________________________________________________________________

Areas of strength of peer research partnership:

____________________________________________________________________________________________________________________

____________________________________________________________________________________________________________________

Areas of peer research partnership to be improved or challenges:

____________________________________________________________________________________________________________________

____________________________________________________________________________________________________________________

Ideas for improvement of peer research partnership:

____________________________________________________________________________________________________________________

____________________________________________________________________________________________________________________

#### 2.3. Version for faculty

This form is intended to help us understand how the peer research partnership program within the FMSTP is working and how it can be improved.

Which peer research partners have you had interaction with? This includes email communication, conference calls, discussion of registrar research progress, etc. Please list all peer mentors you have interacted with: ______________________________________________________________________________________________________________________________________________________________________

What is your overall impression of having peer research partners work with registrars on their research projects?

____________________________________________________________________________________________________________________

____________________________________________________________________________________________________________________

Areas of strength of peer research partnership:

____________________________________________________________________________________________________________________

____________________________________________________________________________________________________________________

Areas of peer research partnership to be improved or challenges:

____________________________________________________________________________________________________________________

____________________________________________________________________________________________________________________

Ideas for improvement of peer research partnership:

____________________________________________________________________________________________________________________

____________________________________________________________________________________________________________________

#### 2.4. Version for administrators

This form is intended to help us understand how the peer research partnership program within the FMSTP is working and how it can be improved.

In what ways have you interacted with the peer research partnership program?

____________________________________________________________________________________________________________________

____________________________________________________________________________________________________________________

What is your overall impression of having peer research partners work with registrars on their research projects?

____________________________________________________________________________________________________________________

____________________________________________________________________________________________________________________

Areas of strength of peer research partnership program:

____________________________________________________________________________________________________________________

____________________________________________________________________________________________________________________

Areas of peer research partnership program to be improved or challenges:

____________________________________________________________________________________________________________________

____________________________________________________________________________________________________________________

Ideas for improvement of peer research partnership program (specific ideas on how to improve the program’s administration and long-term sustainability are welcome):

____________________________________________________________________________________________________________________

____________________________________________________________________________________________________________________

## Appendix 3

### Semi-structured individual interview guides for the Family Medicine Specialty Training Programme trainees, peer mentors, and faculty and administrators

#### 3.1. Version for trainees (referred to below as registrars)

##### Initial perception of programme

1. What do you remember thinking when you first heard about the idea of having a peer partner to work with you on your research?

##### Expectations of the programme

1. What expectations did you have for the peer research partnership programme?
  - How did the programme meet those expectations? In what ways did the programme not meet your expectations?

##### Research curriculum

1. The peer research programme is just one component of the FMSTP research curriculum. Can you tell me about some strengths of the overall research curriculum?
  - Can you give me some examples of ________?
    - Probes: mentorship, support, research skill building?
  1. What are some of the weaknesses of the FMSTP research curriculum?
    - Can you give me some examples of ________?
      - Probes: not enough time f or research, not enough instruction, unclear expectations, lack of organization, confusion between QI, COPC and research requirements ?
2. How has engaging in research during your FMSTP training affected you?
3. What research skills do you feel most confident in?
  - Can you tell me why you feel most confident in _________ skill?
  - How did you peer partner help with this? Can you give me specific examples?
4. What research skills do you feel least confident in?
  - Can you tell me why you feel least confident in _________ skill?
    - Consider choosing specific areas based on most recent survey: choose a research question, conduct a literature review, design a research study, collect and manage research data, analy se research data, write up research present research
5. What do you think are the research mentorship needs of registrars in the FMSTP programme?
  - What role, if any, has your peer partner played in helping meet your own research mentorship needs?

##### Relati onship with your peer partner

1. Tell me a bit about your relationship with your peer partner?
  - What type of things did you learn from them and can you give me some examples?
    - Probe: time management, research principles, etc.
  - In what ways do you feel supported?
    - How did having a peer partner impact your overall stress? How about your overall well-being?
  - How was working with your peer partner?
    - Was there anything challenging about the relationship? If so, can you give me an example of a challenge that you faced with your peer partner
      - Any logistical challenges (network; available time)? Any interpersonal challenges (miscommunication, lack of closeness)?
2. In what ways was your relationship with your peer research partner different from that with your faculty research advisor?
  - With whom did you feel more comfortable asking questions?
3. Imagine you didn’t have a peer partner, how would you feel about completing the FMSTP research requirement?

##### Future

1. How do you see yourself engaging in research in the future?
2. What suggestions do you have for how to improve the FMSTP research curriculum?
  - Was there anything specific that you would have liked to learn but did not get the chance to?
  - Can you explain any specific changes you’d suggest to improve the peer research partnership part of the curriculum?
    - How do you think this programme should continue?
3. We are hoping to build a larger network of peer research mentors by engaging participants of past years to work with future cohorts of trainees as a peer research mentor. What do you think about that idea?
  - How would you feel about being asked to be a peer research mentor?
4. Another idea is to have peer mentors from AfriWon Renaissance support future FMSTP registrars, what do you think about that idea?
  - Any pros/cons you see to that strategy? Can you give me some examples?

##### Demographic verification

1. From programme records, I have the following information that describes you, can we look at this together to ensure this is correct? (Interviewer shows table with demographic information such as year in programme, primary site location, age, gender)

##### Clo sing questions

1. Today you have shared with me about ____, ______ and ______. Taking all this together, is there anything else you’d like to share that would be important for us to know while we work to improve the research experience of FMSTP registrars?

#### 3.2. Version for peer mentors

##### Initial Perception of programme

1. What do you remember thinking when you first heard about the idea of being a peer partner working with a registrar in Lesotho on their research?

##### Expectations

2. What kinds of expectations did you have for the peer research partnership programme?
  - How did the programme meet those expectations? In what ways did the programme not meet those expectations?

##### Research curriculum

3. How do you think engaging in research during the FMSTP training affects registrars?
  - How has this programme affected you?
4. What research skills have you observed the registrars gain while working with them?
  - Can you give me some examples of _______?
5. What research skills do you think they most struggle with?
  - Can you give me some examples of ________?
6. What do you think are the research mentorship needs of registrars in the FMSTP programme?
  - What role, if any, do you think you played in helping meet those research mentorship needs?

##### Relationship/mentorship

7. How would you describe your relationship with the registrar(s) with whom you are paired?
  - Any specific benefits of the relationship? (to you and/or to the registrar)
    - Probes: mutual support, sharing of resources/ideas, new perspectives
  - Any specific challenges you with the relationship? (for you and/or for the registrar)
    (ii) Probes: logistical challenges (network; available time)? interpersonal challenges (miscommunication, role confusion)?
8. Imagine the peer partner programme did not exist, how might that change the registrars experience with completing the FMSTP research requirement?

##### Future

9. What suggestions do you have for how to improve the FMSTP peer research partnership programme?
  - How do you think this programme should continue in future years?
10. What do you think about continuing the model of pairing trainees from the US like you to be peer research partners for FMSTP registrars?
11. Another idea is to engage graduating Lesotho registrars work with future cohorts of trainees as a peer research mentor. What do you think about that idea?
12. Lastly, we are also considering recruiting peer mentors from AfriWon Renaissance, which is the WONCA young doctors’ movement of Africa, support future FMSTP registrars, what do you think about that idea?
  - Any pros/cons you see to this strategy vs the others?
  - Can you give me some examples of how this could be beneficial or not?

##### Demographic verification

13. From programme records, I have the following information that describes you, can we look at this together to ensure this is correct? (Interviewer shows table with demographic information such as year in training, location, gender)

##### Closing questions

14. Today you have shared with me about ____, ______ and ______. Taking all this together, is there anything else you’d like to share that would be important for us to know while we work to improve the research experience of FMSTP registrars?

#### 3.3. Version for faculty/administrators

##### Initial perception of programme

1. What do you remember thinking when you first heard about the idea of having peer partners from the US working with the trainees on their research?

##### Expectations of the programme

1. What kinds of expectations did you have for the peer research partnership programme?
  - How did the programme meet those expectations? In what ways did the programme not meet those expectations?

##### Research curriculum

1. The peer programme is just one component of the FMSTP research curriculum. Can you tell me about some strengths of the overall research curriculum?
  - Can you give me some examples of ________?
  - Probes: mentorship, support, research skill building?
  1. What are some of the weaknesses of the FMSTP research curriculum?
    - Can you give me some examples of ________?
    - Probes: not enough time for research, not enough instruction, unclear expectations, lack of organization, confusion between QI, COPC, and research requirements ?
2. How do you think engaging in research during the FMSTP training affects registrars?
3. What research skills have you observed the registrars gain?
  - Can you tell me why you think they were able to gain that skill?
4. What research skills do you think they most struggle with?
  - Can you tell me why you think they struggle with that skill?
5. What do you think are the research mentorship needs of registrars in the FMSTP programme?
  - What role, if any, do you think the peer partners played in helping meet those research mentorship needs?

##### Relationship

1. Can you share what you observed about the relationship between the peer partners and the registrars?
  - Any specific benefits you observed or were told about?
    - Probes: access to resources, increased skills, decrea sed stress, feeling of support
  - Were there any specific challenges you observed or were told about?
    - Can you give me an example of a challenge?
      - Probes: logistical challenges (network, available time)? interpersonal challenges (miscommunication, r ole confusion)?
2. Imagine the peer partner programme did not exist, how might that change the registrars experience with completing the FMSTP research requirement?

##### Future

1. What suggestions do you have for how to improve the FMSTP research curriculum?
  - Was there anything specific that you would have liked the registrars to learn but did not see done?
  - Can you explain any specific changes you’d suggest to improve the peer research partnership part of the curriculum?
    - How do you think this programme should continue in future years?
2. We are hoping to build a larger network of peer research mentors by engaging participants of past years to work with future cohorts of trainees as a peer research mentor. What do you think about that idea?
3. Another idea is to have peer mentors from AfriWon Renaissance, which is the WONCA young doctors movement of Africa, support future FMSTP registrars, what do you think about that idea?
  - Any pros/cons you see to that strategy? Can you give me some examples of how this could be beneficial or not?

##### Demographic verification

1. From programme records, I have the following information that describes you, can we look at this together to ensure this is correct? (Interviewer shows table with demographic information such as years working for FMSTP, country of origin, gender)

##### Closing questions

1. Today you have shared with me about ____, ______ and ______. Taking all this together, is there anything else you’d like to share that would be important for us to know while we work to improve the research experience of FMSTP registrars?
